# Impact of Systemic Sclerosis–Associated Interstitial Lung Disease, With and Without Pulmonary Hypertension, on Survival: A Systematic Review

**DOI:** 10.7759/cureus.87303

**Published:** 2025-07-04

**Authors:** Shama Shahid Rana, Rana Asjad Dilshad, Shayan Nawaz, Muhammad Usman, Ashna Asif, Farha Jabeen, Malik Aqeel Ahmad, Tauqeer Haider

**Affiliations:** 1 Medicine, Social Security Basic Health Unit (SSBHU) Kanjra, Punjab Employees Social Security Institution (PESSI), Lahore, PAK; 2 General Medicine, Nishtar Medical University, Multan, PAK; 3 Medicine, University College of Medicine and Dentistry, Lahore, PAK; 4 Internal Medicine, Khalid Farooqi Memorial Hospital, Hafizabad, PAK; 5 Pathology, Rashid Latif Medical College, Lahore, PAK; 6 Biochemistry, Abwa Medical College, Faisalabad, PAK; 7 Internal Medicine, Abwa Medical College, Faisalabad, PAK; 8 Medicine, Allied Hospital, Faisalabad, PAK

**Keywords:** dlco, fvc decline, interstitial lung disease (ild), mortality risk, pulmonary arterial hypertension (pah), pulmonary hypertension (ph), survival rate, systemic sclerosis

## Abstract

Many people with systemic sclerosis-associated interstitial lung disease (SSc-ILD) deal with pulmonary hypertension (PH), which seriously reduces their chances of survival. The study aims to evaluate and compare survival outcomes and causes of mortality in patients with SSc-ILD with and without coexisting PH and to assess the implications for clinical management. A comparative review of observational studies and cohort data was conducted to analyze mortality patterns, underlying mechanisms, and treatment responses in SSc-ILD patients stratified by PH status. Based on the Newcastle-Ottawa Scale (NOS) assessment, the overall methodological quality of the included studies was high. Specifically, 16 studies were classified as high quality, with NOS scores ranging from seven to eight, reflecting strong design and execution across the selection, comparability, and outcome domains. Five studies were rated as moderate quality, each scoring six, often due to selection or comparability component limitations. PH commonly affects a significant proportion of patients with SSc-ILD, particularly older adults, individuals of African American ethnicity, and those with reduced diffusing capacity of the lungs. Overall survival tends to be lower in patients with the pulmonary arterial hypertension (PAH) subtype compared to others. A decline in forced vital capacity (FVC) of 10% or more within the first year is linked with higher mortality risk, especially when combined with a marked drop in lung diffusing capacity. Among the various subgroups, patients with both ILD and PH show the poorest five-year survival rates. Important predictors of mortality include the presence of ILD, reduced lung function, and decreased cardiac output. The study concluded that the presence of pulmonary hypertension significantly worsens survival in SSc-ILD patients, primarily through cardiopulmonary failure. These findings highlight the urgent need for early detection, risk stratification, and tailored therapeutic strategies to improve prognosis in this vulnerable population.

## Introduction and background

Systemic sclerosis (SSc) is long-lasting, affects multiple systems, and is characterized by vascular dysfunction, changes in the immune system, and growing scarring throughout the skin and organs [1]. The main contributors to the poor health and death rate associated with this disease are interstitial lung disease (ILD) and pulmonary hypertension (PH) [2]. In approximately 40%-60% of patients with SSc, especially those with diffuse cutaneous SSc, SSc-ILD is likely to happen and may cause pulmonary fibrosis, breathing problems, and lung failure [3]. Similarly, PH of any type of pulmonary arterial hypertension (PAH) in Group 1 or chronic lung disease in Group 3 affects 10%-15% of SSc patients and is linked to a significant reduction in ability and survival [4].

When the conditions coexist, patients with SSc-ILD are at higher risk, and their prognosis can be much worse. Many pathological mechanisms are involved, including how long-term low oxygen in the blood causes the vessels to shrink, planned changes in the blood vessels, and the development of extra scar tissue that blocks the blood vessels [5]. Fibrosis reduces lung flexibility and prevents gas exchange, raising blood vessel pressure and favoring PH development. Unlike hypoxemic respiration, PH increases ventilation-perfusion mismatches, worsening oxygen supply and organ function [6]. This ongoing process results in many patients with both diseases having a much lower survival rate than those with just SSc-ILD, with five-year survival often being as low as 30%-50% [7].

Even though this comorbidity is essential in medical practice, researchers do not fully understand how it impacts survival in SSc-ILD. Current studies have conflicting findings, with a few suggesting that having PH increases the risk of death [8]. In contrast, others indicate that severe ILD itself may be the primary driver of poor outcomes [9]. These discrepancies likely stem from several factors: variability in PH diagnostic criteria (right heart catheterization versus echocardiography), differing hemodynamic thresholds (mean pulmonary arterial pressure (mPAP) >20 mmHg versus >25 mmHg), heterogeneous study populations, and inconsistent adjustment for ILD severity and other confounding factors [8, 9]. Furthermore, the prognostic significance of PH may vary depending on SSc subtype, autoantibody profiles, and the timing of PH development in the disease course [10].

Given these uncertainties and the high mortality associated with SSc-ILD-PH overlap, a comprehensive systematic review is urgently needed to clarify several key aspects which are as follows: (1) the magnitude of the survival difference between SSc-ILD patients with versus without PH, (2) whether PH represents an independent risk factor when accounting for ILD severity, (3) how different diagnostic approaches influence prognostic estimates, and (4) which clinical or demographic factors modify this relationship. By synthesizing existing evidence, this review will provide clinicians with crucial insights for risk stratification, guide therapeutic decision-making, and highlight critical knowledge gaps to direct future research efforts to improve outcomes for this vulnerable patient population. The findings will be particularly relevant in emerging therapies targeting both fibrotic and vascular pathways in SSc, helping optimize treatment strategies for patients with this devastating disease combination.

## Review

Method

This review followed the requirements established by Preferred Reporting Items for Systematic reviews and Meta-Analyses (PRISMA) guidelines [11]. The research question was formulated using the Population, Intervention, Comparison, and Outcome (PICO) framework in Table 1 [12].

**Table 1:** Population, Intervention, Comparison, and Outcome (PICO) framework MeSH: Medical Subject Headings

Concepts	Text words	Controlled vocabulary
Population: Problem patients with systemic sclerosis-associated interstitial lung disease (SSc-ILD)	“Systemic Sclerosis,” “Scleroderma,” “Interstitial Lung Disease,” “Pulmonary Fibrosis,” “SSc-ILD”	"Scleroderma, Systemic"[MeSH], "Lung Diseases, Interstitial"[Mesh]
Intervention: Presence of pulmonary hypertension	“Pulmonary Hypertension,” “SSc-ILD with PH,” “Elevated Pulmonary Pressure”	"Hypertension, Pulmonary"[MeSH]
Comparison: Absence of pulmonary hypertension	“Without Pulmonary Hypertension,” “SSc-ILD alone,” “Normotensive SSc-ILD”	Not directly a MeSH term; use context or filtering
Outcomes: Survival outcomes	“Survival,” “Mortality,” “Prognosis,” “Life Expectancy,” “Overall Survival”	"Survival"[MeSH], "Mortality"[MeSH], "Prognosis"[MeSH]

Research Question

What is the impact of SSc-ILD with and without PH on survival?

Search Strategy and Search Terms

Searches were conducted using combinations of keywords and controlled vocabulary related to "systemic sclerosis," "interstitial lung disease," "pulmonary hypertension," and survival outcomes such as "mortality," "prognosis," and "life expectancy." Boolean operators "AND" and "OR" were used to connect relevant terms across databases, including PubMed, the Excerpta Medica database (Embase), and the Cochrane Library. The search focused on text words and controlled vocabulary (e.g., Medical Subject Headings (MeSH) terms) to ensure comprehensive coverage. Limiters were applied to retrieve only open-access, full-text articles published in English between 2014 and 2024 and studies involving human participants.

Search String

("Systemic Sclerosis" OR "Scleroderma" OR "SSc") AND ("Interstitial Lung Disease" OR "SSc-ILD" OR "Pulmonary Fibrosis") AND ("Pulmonary Hypertension" OR "PH" OR "Elevated Pulmonary Artery Pressure") AND ("Survival" OR "Mortality" OR "Prognosis" OR "Life Expectancy").

Inclusion Criteria

Experimental studies, cohort, case-control, and observational studies with varying sample sizes were included in this review. Studies in which adult patients of either gender with SSc-ILD, with and without PH, on survival were included. The studies focused on survival, mortality, prognosis, life expectancy, and overall survival. The synthesis included studies from the last two decades that were open access, written in English, and available in full text.

Exclusion Criteria

The review excluded all other study designs, case reports, case series, conference abstracts, editorials, letters, review papers, and meta-analyses. Studies on teenagers, children, and animals were also excluded. Studies before 2005 in which patient outcomes were not related to the impact of SSc-ILD with and without PH on survival were excluded due to restricted data access and incomplete analysis.

Study Selection Process

The initial screening involved two independent reviewers reading the articles' titles and abstracts. Then, the two independent reviewers conducted a full-text review by comprehensively reading the articles. Regarding reviewers' disagreement, a consensus was developed [13]. The review included only those studies available in full text that met the inclusion criteria.

Methodological Quality Assessment

The Newcastle-Ottawa Scale (NOS) classifies the quality of non-randomized studies into three categories based on a star rating system, with a maximum of nine stars awarded across three domains: selection, comparability, and exposure or outcome. Studies scoring between seven and nine stars are generally considered high quality, reflecting a low risk of bias and intense methodological rigor. Those with five to six stars are rated as moderate quality, indicating some potential for bias but generally acceptable design. Scores of 0 to four stars denote low-quality studies, which may have significant methodological weaknesses and a higher risk of bias [14].

Data Extraction and Synthesis

A datasheet was created to collect details about the data we needed to extract from the included studies to synthesize study findings. In terms of the current study, it encompassed basic information, such as study design, demographic characteristics, and characteristics related to the outcome of interest, including author/year, objectives, study design/population characteristics, treatment, outcome measured, survival analysis, study findings, prognostic factors, and complications. After that, a thematic analysis using an inductive, data-driven approach was employed to analyze the data sheet [15]. Then, an iterative approach was applied for a further in-depth study and convergence of the results [16]. Then, studies are analyzed critically to synthesize the evidence, ensuring the practice is evidence-based.

Ethical Consideration

**Figure 1:**
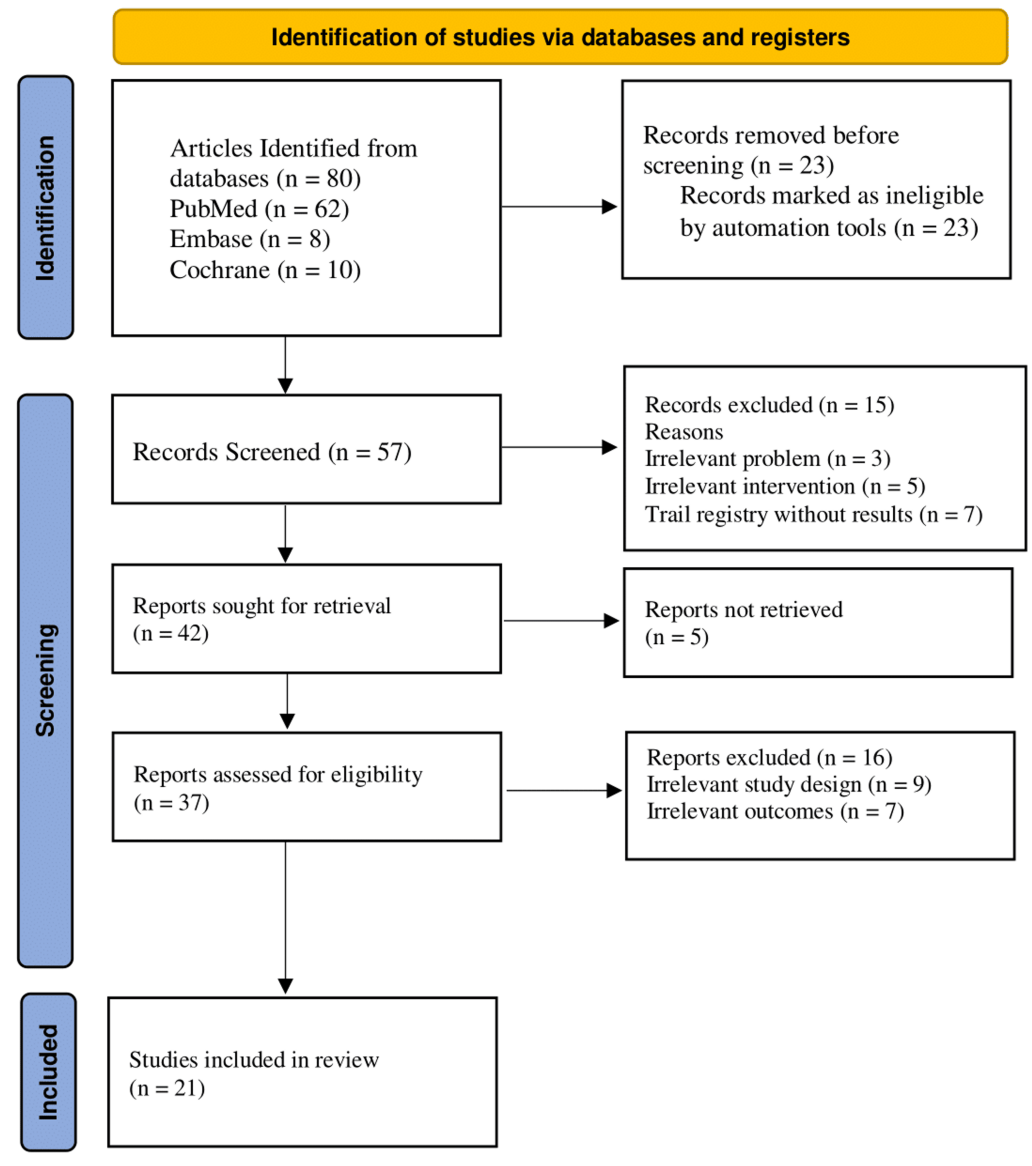
A PRISMA flowchart outlining the study selection process PRISMA: Preferred Reporting Items for Systematic Reviews and Meta-Analyses; Embase: Excerpta Medica database;

The review adhered to the Helsinki Declaration to meet ethical standards throughout the study. There are no conflicts of interest among the reviewers. It was performed with specific keywords to reproduce it. The study will be published in a medical journal to disseminate findings publicly while ensuring confidentiality and anonymity. The study met the PRISMA guidelines (Figure 1).

Results

The PRISMA guidelines were followed to synthesize the evidence in this review systematically. The 80 articles were retrieved during the initial search using keywords, text words, and controlled vocabulary on PubMed, Embase, and the Cochrane Library databases. The 23 duplicate articles were removed using EndNote (Clarivate, Philadelphia, PA). Then, 57 articles were selected for screening. Then, 15 irrelevant articles were removed based on title and abstract reading. Forty-two articles were requested for retrieval, but five were removed due to unretrieved reports. The eligibility of 37 articles was determined through pre-specified criteria. Furthermore, 16 irrelevant articles were excluded through a thorough and in-depth review of the studies. After the eligibility check, only 21 articles were selected for quality assessment.

Quality Assessment of Studies in the Systematic Review

The NOS classifies the quality of non-randomized studies into three categories based on a star rating system, with a maximum of nine stars awarded across three domains: selection, comparability, and exposure or outcome. Studies scoring between seven and nine stars are generally considered high quality, reflecting a low risk of bias and strong methodological rigor. Studies with a rating of five to six stars are considered moderate quality because they might have a little bias, but are generally fine in design. When a study gets 0 to four stars, it’s usually low quality and may be biased due to flawed methods. Table 2 shows the NOS assessment of the studies

**Table 2:** Newcastle-Ottawa Scale (NOS) assessment of the studies

Study	Selection	Comparability	Outcome	Total score	Quality
Young et al., 2019 [17]	3	1	3	7	High quality
Hoffmann-Vold et al., 2021 [18]	3	2	3	8	High quality
Goh et al., 2017 [19]	3	1	3	7	High quality
Kolstad et al., 2018 [20]	2	1	3	6	Moderate quality
Launay et al., 2013 [21]	2	2	2	6	Moderate quality
Rubenfire et al., 2013 [22]	3	1	2	7	High quality
Moinzadeh et al., 2024 [23]	3	2	3	8	High quality
Guillén-Del-Castillo et al., 2022 [24]	3	2	2	7	High quality
Amikishiyev et al., 2025 [25]	3	2	2	7	High quality
Chan et al., 2025 [26]	3	2	3	8	High quality
Fairley et al., 2023 [27]	3	2	2	7	High quality
Noviani et al., 2019 [28]	2	1	3	6	Moderate quality
Mathai et al., 2009 [29]	2	1	3	6	Moderate quality
Guler et al., 2018 [30]	3	1	3	7	High quality
Chauvelot et al., 2020 [31]	3	2	3	8	High quality
Cacciapaglia et al., 2023 [32]	3	2	2	7	High quality
Le Pavec et al., 2011 [33]	3	2	2	7	High quality
Trad et al., 2006 [34]	3	2	2	7	High quality
Bauer et al., 2013 [35]	3	2	3	8	High quality
Jang et al., 2023 [36]	3	1	3	7	High quality
Knarborg et al., 2022 [37]	2	2	2	6	Moderate quality

Applying the NOS assessment, most of the included studies showed a high methodology standard, with total marks between seven and eight out of nine. For instance, Hoffmann-Vold et al. (2021), Moinzadeh et al. (2024), Chan et al. (2025), Chauvelot et al. (2020), and Bauer et al. (2013), along with other studies, performed very well (scoring eight) on all the key criteria [18, 23, 26, 31, 35]. Several studies, including Young et al. (2019), Goh et al. (2017), and Guler et al. (2018), scored seven, indicating high quality with minor limitations [17, 19, 30]. In contrast, a smaller subset of studies, such as Kolstad et al. (2018), Launay et al. (2013), and Knarborg et al. (2022), were categorized as moderate quality with scores of six, often due to lower ratings in the selection or comparability domains [20, 21, 28, 29, 37]. Overall, the included literature presents predominantly high-quality evidence, supporting the robustness of the findings in the context of non-randomized study evaluation.

Characteristics and Findings of Studies Included in the Review

Table 3 describes the characteristics and findings of studies included in the review. It encompasses author/year, objectives, study design/population characteristics, treatment, measured outcomes, survival analysis, study findings, prognostic factors, and complications.

**Table 3:** Characteristics and findings of studies included in the review 6MWD: 6-minute walk distance; AZA: azathioprine; BMI: body mass index; CCD: composite clinical decline (i.e., FVC + DLCO decline); CI: confidence interval; CRP: C-reactive protein; CYC: cyclophosphamide; dcSSc: diffuse cutaneous systemic sclerosis; DLCO / DLco: diffusing capacity of the lung for carbon monoxide; ePASP: estimated pulmonary artery systolic pressure; ERA: endothelin receptor antagonist (e.g., bosentan); ESR: erythrocyte sedimentation rate; FVC: forced vital capacity; GAP index: gender, age, physiology; HR: hazard ratio; HRCT: high-resolution computed tomography; HRQoL: health-related quality of life; ILD: interstitial lung disease; IQR: interquartile range; Kco: transfer coefficient of the lung for carbon monoxide; lcSSc: limited cutaneous systemic sclerosis; MMF: mycophenolate mofetil; mPAP: mean pulmonary arterial pressure; mRSS: modified Rodnan skin score; MTX: methotrexate; NSIP: nonspecific interstitial pneumonitis; NYHA: New York Heart Association functional class; OR: odds ratio; PAH: pulmonary arterial hypertension; PCA: prostacyclin analog (e.g., epoprostenol); PDE5i: phosphodiesterase-5 inhibitor (e.g., sildenafil); PFT: pulmonary function test; PH: pulmonary hypertension; PH-ILD: pulmonary hypertension associated with interstitial lung disease; PVOD: pulmonary veno-occlusive disease; PVR: pulmonary vascular resistance; RAP: right atrial pressure; RHC: right heart catheterization; RVSP: right ventricular systolic pressure; SSc: systemic sclerosis; SSc-ILD: systemic sclerosis-associated interstitial lung disease; SSc-PAH: systemic sclerosis-associated pulmonary arterial hypertension; SMR: standardized mortality ratio; SRC: scleroderma renal crisis; TAPSE: tricuspid annular plane systolic excursion; TTCW: time to clinical worsening; WHO-FC: World Health Organization functional class

Author / Year	Objectives	Study design/Population characteristics	Treatment	Outcome measured	Survival analysis	Study findings	Prognostic factors	Complications
Young et al., 2019 [17]	Assess PH prevalence, features, therapy, and outcomes in SSc-ILD; evaluate PH survival and predictors	Prospective cohort; N = 93 with SSc-ILD PH present in 29 (31.2%), confirmed via RHC. Mean age 54.9; 76% female; 84.9% White, 8.6% African American	ILD: 82.8% received immunosuppressants (MMF, CYC, rituximab). PAH: 82.8% received PAH-targeted therapy - Monotherapy: PDE5i or ERA - Combination: 31% dual (PDE5i + ERA), 13.8% triple (incl. prostacyclins)	PH prevalence: 31.2% ILD subtype: 90.3% NSIP on HRCT FVC: 76.2% predicted DLCO: 58.3% predicted	Overall survival: 97% at 3 years (total cohort). PH subgroup: 91% at 3 years after PH diagnosis, 3 total deaths (2 in PH group: 1 PH progression, 1 infection)	PH more frequent in older patients (55.8 vs. 49.7 yrs, p=0.02), Higher proportion of African Americans in PH group (20.7% vs. 3.1%, p=0.02), Lower DLCO (46.8% vs. 63.4%, p<0.001)	Older age at ILD diagnosis, African American race, Lower DLCO, Higher FVC/DLCO ratio	6.9% mortality in the PH group 31% required supplemental oxygen
Hoffmann-Vold et al., 2021 [18]	To identify the disease course, progression patterns, and risk factors for progressive SSc-ILD.	Prospective cohort study; N = 826 with SSc-ILD. Mean age: 56 years; 82% female. 50% diffuse, 50% limited cutaneous SSc.	37% received immunosuppressants (MMF, CYC, MTX). Antifibrotic: Nintedanib was approved during the study.	ILD progression: FVC decline ≥5%. Disease heterogeneity: decline/stability periods.	10% mortality over follow-up. 48% stable, 25% improved, 27% progressed.	ILD progression was variable and unpredictable. Rapid decline in 8%, slow progression in 58%.	Male sex, reflux/dysphagia, high mRSS, elevated ESR. Paradoxical association with higher baseline FVC.	Delayed treatment after FVC decline may lead to irreversible damage.
Goh et al., 2017 [19]	To assess the prognostic value of short-term PFT trends in SSc-ILD and identify thresholds predicting long-term mortality.	Retrospective cohort study; N = 162 SSc-ILD patients from an initial 215 (exclusions: death within 9 months or lack of serial PFTs). Mean age: 48 years; 82% female.	46% received immunosuppressants (not adjusted for in final analysis).	PFT trends: 1-year: FVC decline ≥10% or composite decline (FVC 5–9% + DLCO ≥15%), 2-year: DLCO and KCO changes.	15-year survival analysis. Overall mortality: 52% (84/162) over median 155 months.	The CCD was the strongest predictor of mortality. FVC decline ≥10% at 1 year was associated with increased risk (HR = 1.84, p = 0.01).	Extensive fibrosis (HR = 3.01, p < 0.0005), Low DLCO and KCO, Older age, DLCO decline ≥15% (HR = 2.03, p < 0.005), KCO decline ≥10% (HR = 2.35, p < 0.001)	No specific complications reported; immunosuppressant effects not controlled in final analysis.
Kolstad et al., 2018 [20]	To assess long-term outcomes and predictors of mortality in patients with SSc-PAH.	Prospective cohort study; N = 160 incident SSc-PAH patients. 89.3% female; mean age 60.0 ± 10.9 years. 71% had limited cutaneous SSc. NYHA class I (16%), II (44%), III/IV (40%).	Initial therapies: PDE5 inhibitor monotherapy (57%), ERA monotherapy, Oral combination therapy, parenteral prostacyclin.	Primary outcomes: all-cause and PAH-related mortality. Secondary: TTCW – includes death, hospitalisation, lung transplant, worsening symptoms.	Overall survival: 1 year: 95%, 3 years: 75%, 5 years: 63%, 8 years: 49% TTCW: 70% by 8 years. 93% of PAH-related deaths occurred within 4 years.	PAH was the primary cause of death in 61% of early deaths. Clinical worsening occurred in the majority by year 8.	Male sex (HR 3.11), Diffuse SSc (HR 2.12), Low DLCO (HR 0.65), Shorter 6MWD (HR 0.92), Higher mPAP (HR 1.03) and PVR (HR 1.11), Better prognosis with NYHA I/II and higher cardiac output.	PAH progression, SSc-related organ failure, cancer, and infection contributed to mortality; 52% of total deaths were PAH-related.
Launay et al., 2013 [21]	To assess survival and prognostic factors in incident SSc-PAH patients without ILD in the modern treatment era.	Prospective cohort study; N = 85 incident SSc-PAH patients (without ILD). 82% female; mean age 64.9 ± 12.2 years. 87% had limited cutaneous SSc.	First-line therapies: ERA monotherapy (69%, mostly bosentan), PDE5 inhibitors (16%), Combination therapy (5%), epoprostol (1%). Follow-up: 26 received ERA + PDE5i; 18 received prostacyclins.	Primary: all-cause mortality. Secondary: survival at 1, 2, and 3 years.	Overall survival: 1 year: 90%, 2 years: 78%, 3 years: 56% 2 transplant recipients remained alive at follow-up.	Clinical worsening occurred early despite modern therapies. High 3-year mortality rate. PAH progression was the leading cause of death.	Older age (HR 1.05, 95% CI 1.01–1.09, p=0.012), Low cardiac index (HR 0.49, 95% CI 0.27–0.89, p=0.019), Male gender (not statistically detailed)	PAH progression was the primary cause of death. Suspected PVOD in 2 transplant patients. Non-significant predictors: 6MWD, mPAP, RAP.
Rubenfire et al., 2013 [22]	To compare clinical/hemodynamic variables, treatments, and survival in SSc-PAH vs. idiopathic/familial/anorexigen-associated PAH (PAHifa).	Retrospective cohort study. SSc-PAH: N = 83; mean age 59; 79.5% female; 84.3% NYHA FC III/IV. PAHifa: N = 120; mean age 51; 85% female; 80% NYHA FC III/IV.	SSc-PAH: 44% received prostacyclins. Similar use of ETAs and PDE5 inhibitors across both groups. PAHifa: 71% prostacyclin use.	Primary: all-cause mortality. Secondary: mPAP, PVR, cardiac index, 6MWD, functional class.	SSc-PAH survival: 1 year: 84%, 3 years: 60%, 5 years: 51% PAHifa survival: 1 year: 99%, 3 years: 88%, 5 years: 87% Log-rank test: P < .001	SSc-PAH had worse survival than PAHifa despite more favorable hemodynamics. Right ventricular dysfunction and myocardial fibrosis were implicated.	Male sex in SSc-PAH (HR = 2.98, p = .024), Higher FVC/DLCO ratio (HR = 2.40, p = .041), PAHifa: 6MWD < 250 m (HR = 5.23, p = .017)	No observed survival benefit in SSc-PAH from modern therapies. Potential role of PVOD in SSc-PAH mortality.
Moinzadeh et al., 2024 [23]	To determine the prevalence, clinical characteristics, and survival of SSc-associated ILD with and without PH.	Large-scale cohort study; N = 3,257 SSc patients. Mean follow-up: 3.45 ± 1.63 years.	ILD patients: Glucocorticoids (58.1% ILD-PH, 54.0% ILD w/o PH), Immunosuppressants (67.7% ILD-PH, 70.9% ILD without PH)	Prevalence and impact of ILD and PH. Kaplan-Meier survival estimates.	ILD-PH group: 5-year survival = 79.1%, ILD without PH: 92.8%, PAH alone: 85.0%, No lung involvement: 96.4% ILD-PH had the highest mortality risk (HR = 5.3, p < 0.001)	ILD-PH and PAH were associated with significantly worse outcomes. Progressive fibrosis and inflammation (elevated CRP, ESR) were more common in ILD-PH.	Negative: ILD-PH, PAH, male sex, low BMI, low DLCO; Protective: Female sex (HR = 0.3), higher BMI (HR = 0.9), higher DLCO (HR = 0.98)	No treatment-related complications were detailed. Disease progression was linked to the diffuse cutaneous SSc subtype and inflammation.
Guillén-Del-Castillo et al., 2022 [24]	To assess severity markers and outcomes in SSc patients with or without PAH, and examine the impact of ILD on PAH-SSc outcomes.	Retrospective cohort study. PAH-SSc: N = 364 (86.8% female; mean age 62.7 years). Non-PAH-SSc: N = 1589 (88.6% female; mean age 51.3 years). ILD prevalence: 41.8% (PAH-SSc) vs. 44.9% (non-PAH-SSc).	PAH-SSc: 62.6% received up-front combination therapy, 32.7% monotherapy. Non-PAH-SSc: 15.9% received vasodilators (for vasculopathy).	Transplant-free survival, hemodynamics, PFTs, NYHA class, echocardiography.	5-year survival: , PAH-SSc: 41.1% , Non-PAH-SSc: 93.9% P < 0.001 No survival difference between PAH-SSc with vs. without ILD (P = 0.444).	PAH-SSc had more severe hemodynamic compromise and poorer functional status. ILD worsened lung function but did not affect survival significantly.	Older age (HR = 1.02, P = 0.036) , NYHA class III-IV (HR = 1.63, P = 0.015) , Higher PVR (HR = 2.41, P = 0.002) , Better: Higher DLCO (HR = 0.87, P = 0.009) , Better: Up-front combo therapy (HR = 0.54, P < 0.001)	Higher pericardial effusion in PAH-SSc (30% vs. 5.2%, P < 0.001), Worse TAPSE in PAH-SSc (17.4 mm vs. 19.9 mm, P < 0.001), ILD in PAH-SSc: Lower FVC and DLCO, but not linked to excess mortality.
Amikishiyev et al., 2025 [25]	To investigate mortality and prognostic factors in SSc patients with PH, with or without ILD.	Retrospective cohort study; N = 44 SSc-PH patients. 42 female; mean age 56.6 ± 13.5 years. Diagnosed by right heart catheterization. Follow-up: 2008–2022, Turkey.	PAH-specific therapy: Monotherapy: 70.5%, Dual therapy: 25%, 34% eventually switched to combined therapy.	Mortality, echocardiographic and RHC parameters, pulmonary function tests, and treatment modalities.	Survival rates: 1 year: 91%, 2 years: 75%, 5 years: 43.1% Median survival: Monotherapy: 44 months, Combined therapy: 61 months (P = .01)	The PH + ILD group had significantly higher mortality (P = .007). Deceased patients had higher ePASP, lower cardiac output, and reduced FVC (P = .01–.02).	ILD (OR = 5.7, P = .008), Low FVC (OR = 5.3, P = .01), Low cardiac output (OR = 2.3, P = .03), Worse WHO functional class, and monotherapy are associated with poor prognosis.	Causes of death: cardiopulmonary failure (26%), infections (26.1%, incl. COVID-19), malignancy (13%). Higher ePASP, low FVC, and cardiac output in deceased patients.
Chan et al., 2025 [26]	To determine the prevalence of ILD in SSc, identify risk factors for ILD development, and explore prognostic factors in SSc-ILD.	Retrospective cohort study; N = 223 SSc patients. 86.1% female; median age at diagnosis: 55 years. Subtypes: 71.3% limited cutaneous SSc (lcSSc), 28.7% diffuse cutaneous SSc (dcSSc). Median follow-up: 8.1 years (IQR: 4.0–10.2).	Immunosuppressants (e.g., MMF, cyclophosphamide) are likely used, though treatment effects are confounded by indication. Antifibrotic agents (e.g., nintedanib) are suggested as alternatives.	ILD prevalence, progression, mortality, pulmonary function (FVC, DLCO), and inflammatory markers (CRP, ESR).	Overall mortality: 24.2%, ILD group: 29.7%, Non-ILD group: 18.8% (P = 0.056). Significant survival difference between groups (P < 0.05, log-rank test).	ILD prevalence: 49.8% (111/223). Among ILD patients, 64.1% showed disease progression. Progressive ILD was associated with worse pulmonary function and mortality.	Bibasal crackles (HR = 2.813, P = 0.001), CRP at diagnosis (HR = 1.103, P = 0.009), Smoking (HR = 5.173, P = 0.028), Progressive ILD trend: HR = 2.823 (P = 0.097)	Pneumonia was the most common cause of death (63.6% in the ILD group). , PH more frequent in ILD patients (19.8% vs. 2.7%, P < 0.001), Decline in lung function: FVC (70.0% vs. 85.2%), DLCO (43.3% vs. 62.3%).
Fairley et al., 2023 [27]	1. Describe the clinical phenotype and prognosis of SSc patients with PAH with/without ILD. 2. Compare survival and HRQoL among PAH-ILD, PAH-only, ILD-only, and SSc-only groups.	Cohort study; N = 1561 SSc patients. 86% female; 74% had limited cutaneous SSc (lcSSc), 26% diffuse cutaneous SSc (dcSSc). Group breakdown: PAH-ILD (7%, n = 107), PAH-only (7.2%, n = 112), ILD-only (23.9%, n = 372), SSc-only (62.1%, n = 970).	PAH-specific therapy: ERA most common, PDE5 inhibitors, parenteral prostanoids, 90.7% of PAH-ILD and 98.2% of PAH-only received PH-specific meds (P = 0.014), Immunosuppressants more frequent in ILD groups (e.g., MMF, cyclophosphamide, prednisolone; P < 0.001).	Primary: All-cause mortality. Secondary: HRQoL (SF-36, Scleroderma Health Assessment Questionnaire, PFTs (FVC, DLCO), WHO class, 6MWD, autoantibodies.	Mortality: PAH-ILD: 50.5% deceased vs. 6.7% in SSc-only (P < 0.001), PAH-ILD: HR = 5.68 (95% CI: 3.51–9.17, P < 0.001), PAH-only: HR = 4.30; extensive ILD-only: HR = 3.72	PAH-ILD had the worst survival and lowest lung function (FVC 63%, DLCO 33.4%). PAH-ILD was associated with older onset, male sex, dcSSc, higher CRP/ESR, and Asian ethnicity (P < 0.001).	Poor prognosis: Extensive ILD + PAH (HR = 5.68), PAH-only (HR = 4.30), SRC history (HR = 2.60, P = 0.001), Older age at onset (HR = 1.11, P < 0.001), Extensive ILD-only (HR = 3.72), Protective: ANA centromere (HR = 0.65), female sex (HR = 0.61)	Infections were the leading cause of death. GI symptoms (dysphagia, vomiting) were more frequent in PAH groups (P < 0.02), SRC was higher in PAH-ILD vs. PAH-only (6.5% vs. 0.9%, P = 0.040), HRQoL: PAH-ILD had the worst scores (OR = 12.48 for poor SF-36 PCS, P < 0.001).
Noviani et al., 2019 [28]	To compare mortality and hospitalization rates among SSc patients with isolated PAH, isolated ILD, concomitant ILD-PH, and no/mild pulmonary involvement.	Retrospective cohort study; N = 490 SSc patients. 87% female; mean age 47.6 ± 14.6 years. Groups: ILD-PH, PAH, ILD, and no/mild pulmonary involvement.	PAH-specific medicines were used in 62% of PAH and 60.5% of ILD-PH patients. Immunosuppressants (MTX, CYC, MMF) were used in 50–69.6% of patients.	Primary: All-cause mortality and hospitalization rate (mean admissions/year).	5-year survival from SSc diagnosis: , ILD-PH: 77% , PAH: 88% , ILD: 90% , No/mild lung disease: 93% (P < 0.001) From PH/ILD diagnosis: , ILD-PH: 76.9% , PAH: 67.5% , ILD: 87.2% (P = 0.009)	ILD-PH had the highest unadjusted mortality risk (HR = 3.77, P < 0.001). PAH independently predicted mortality (HR = 2.39, P = 0.023). Hospitalizations were highest in PAH (β = 0.41) and ILD-PH (β = 0.39), but were not significant after adjustment (P = 0.136).	, Male gender (HR = 3.02, P = 0.002) , Older age at diagnosis (HR = 1.06, P < 0.001) , Malabsorption (HR = 2.59, P = 0.001) , PAH medication use associated with hospitalization (β = 0.22, P = 0.003)	Leading causes of death: PAH and ILD (34%), Main drivers of hospitalization: renal crisis, right heart failure, PAH therapies.
Mathai et al., 2009 [29]	1. Compare survival between SSc patients with PAH and ILD-associated PH. 2. Identify survival predictors in PH-SSc patients receiving PAH-specific therapy.	Retrospective cohort study; N = 59 SSc-PH patients confirmed via right heart catheterization. PAH group: n = 39 ILD-associated PH group: n = 20 88% female; 78% had limited cutaneous SSc.	PAH-specific therapy: ERA (71%, mostly bosentan/sitaxsentan), PDE5i (10%, sildenafil), Prostanoids (17%, epoprostenol). Immunosuppressants were used in 70% of ILD-PH patients (prednisone, cyclophosphamide, MMF).	Primary: Transplant-free survival. Secondary: Hemodynamics (PVR, mPAP, cardiac index), WHO class, pulmonary function (FVC, DLCO).	3-year survival: ILD-PH: 82% (1-year), 46% (2-year), 39% (3-year), PAH: 87% (1-year), 79% (2-year), 64% (3-year), P < 0.01	ILD-PH patients had lower FVC (54%) and DLCO (31%). Despite PAH therapy, the ILD-PH group had worse survival PAH group had higher mPAP (49 vs. 40 mmHg). Cause of death: ILD-PH, respiratory failure (75%), PAH – right heart failure (61%)	ILD-associated PH (HR = 5.15, P < 0.01) , Lower cardiac index (HR = 0.41, P = 0.04) , Lower DLCO in ILD-PH (HR = 0.93, P = 0.02) , Higher PVR index (HR = 1.05, P < 0.01)	Severe hypoxemia in ILD-PH (60% required oxygen), High mortality despite immunosuppression and PAH therapy
Guler et al., 2018 [30]	1. Determine whether the observed plateau in pulmonary function decline in SSc-ILD is due to survival bias. 2. Identify prognostic subgroups of ILD progression.	Prospective cohort study; N = 171 patients with SSc-ILD. Mean age: 55.1 years; 83% female; 44% ever-smokers.	Immunosuppressive therapy initiated based on ILD worsening: cyclophosphamide, azathioprine, mycophenolate mofetil	Primary: Annual rate of FVC and DLCO decline (% predicted). Secondary: Survival analysis; predictive value of prior year’s FVC/DLCO trend.	Patients with short-term mortality showed steeper declines: , FVC: −4.10% vs. −0.94% per year (P = 0.003) , DLCO: −5.28% vs. −1.32% (P < 0.001)	No plateau in pulmonary decline was observed after accounting for survival bias. DLCO trends were predictive of future DLCO decline (P = 0.02). FVC trend did not predict future change (P = 0.08). Prognostic phenotypes based on lung function trajectory were identified.	Poor prognosis: rapid FVC/DLCO decline, short-term mortality. Better prognosis: slow decline, long-term survival. Non-significant: baseline RVSP, anti-centromere status.	No major treatment complications reported; focused on physiological decline and survival trajectories.
Chauvelot et al., 2020 [31]	To compare survival and treatment response in SSc patients with PH-ILD versus isolated PAH.	Prospective cohort study; N = 128 SSc patients. 66 with PH-ILD; 62 with PAH only.	PAH-specific therapy: ERA: 84.9% (PH-ILD) vs. 72.6% (PAH), PDE5 inhibitors: 10.6% (PH-ILD) vs. 14.5% (PAH), Prostacyclin analogs: 3.0% (PH-ILD) vs. 1.6% (PAH), Sequential oral combination therapy in 15 (PH-ILD) and 22 (PAH) patients. Immunosuppressive therapy was administered, particularly in PH-ILD.	Hemodynamic parameters (mPAP, PVR, cardiac index), 6MWD, WHO functional class, oxygen therapy initiation, and mortality.	1-/2-/3-year survival: PH-ILD: 8.1%, 21.2%, 41.5%, PAH: 4.1%, 8.7%, 21.4% P = 0.04 (log-rank test). PH-ILD had significantly worse survival (HR = 2.11, P = 0.01).	PH-ILD patients showed less WHO class improvement (13.6% vs. 33.3%, P = 0.02). No significant differences in hemodynamic improvement (ΔPVR, P = 0.47) or 6MWD (P = 0.11). Oxygen therapy initiation was higher in PH-ILD (27.4% vs. 15.8%, P = 0.13).	ILD (HR = 2.11, P = 0.01) , Chronic kidney disease (HR = 5.22), Lower 6MWD (HR = 0.996), Immunosuppression reduced mortality in PH-ILD (HR = 0.46, P = 0.02)	PH-ILD patients were more likely to require oxygen therapy. No significant worsening of oxygenation due to PAH-targeted therapies.
Cacciapaglia et al., 2023 [32]	To update 5- and 10-year survival rates and identify prognostic factors in Italian SSc patients diagnosed after 2009.	Retrospective cohort study; N = 912 SSc patients. 91.6% female; 20% had diffuse cutaneous SSc.	Glucocorticoids (40.8%), Immunosuppressants (35.3%), Biologic agents (7.9%), Vasoactive drugs (e.g., ERA, PDE5 inhibitors)	Overall survival, 5- and 10-year survival, standardized mortality ratio (SMR), predictors of mortality (Cox regression).	5-year survival: 94.4%, 10-year survival: 89.4%, SMR: 0.96 (95% CI: 0.81–1.13). Survival was significantly reduced in PAH and ILD-PH groups (P < 0.0001), but not in isolated ILD (P = 0.23).	Significant improvement in long-term survival compared to historical cohorts (e.g., 32% in the 1980s). Early diagnosis (within 2 years of Raynaud’s onset) was linked to improved outcomes.	Male gender (OR = 2.76, P = 0.008), Older age at diagnosis (OR = 1.08 per year, P < 0.001), and PAH or ILD-PH were associated with significantly lower survival.	Comorbidities reported: hypertension (28.2%), diabetes (5.1%), CKD (6.7%). No treatment-related complications were highlighted.
Le Pavec et al., 2011 [33]	To evaluate the efficacy and safety of PAH-specific therapies in systemic sclerosis (SSc)-related pulmonary hypertension (PH) associated with interstitial lung disease (ILD).	Retrospective study; N = 70 SSc patients with PH-ILD confirmed by right heart catheterization (RHC). Mean age: 57.2 years 50% had limited cutaneous SSc 85% were ANA-positive.	All patients received PAH-specific therapy: Endothelin receptor antagonists (66%), PDE5 inhibitors (29%), Prostacyclin analogs (5%), 31% received second- or third-line agents.	WHO functional class, 6MWD, hemodynamics (mPAP), oxygenation, survival.	Survival rates: 1 year: 71%, 2 years: 39%, 3 years: 21% 33 deaths and 3 lung transplants occurred during a mean follow-up of 24 months.	No significant improvement in WHO functional class (P = 0.52), 6MWD (P = 0.77), or hemodynamics (mPAP P = 0.63). Oxygenation decline and renal dysfunction predicted poor survival.	Worsening oxygenation (HR = 3.11, P = 0.04), reduced eGFR (HR = 0.54, P = 0.03) Poor prognostic factors identified in multivariate analysis.	Worsening oxygenation (24 patients, P < 0.001), increased oxygen requirements. Causes of death: respiratory failure (48%), right-sided heart failure (30%), cancer, mesenteric ischemia, sudden death
Trad et al., 2006 [34]	1. Determine if PAH is an independent prognostic factor for mortality in dcSSc, regardless of ILD. 2. Evaluate CYC efficacy in treating ILD and PAH.	Retrospective cohort study; N = 86 DC-SSc patients. Mean age: 44.5 years; 88% female; 70% anti-Scl-70 positive.	Immunosuppressive therapy: 55% received steroids and/or CYC, CYC IV monthly pulses (6–21 doses; mean 10) in the ILD group, the PAH group more frequently received anticoagulants (P = 0.005)	Primary: Mortality predictors (PAH, ILD, age) Secondary: PFTs (TLC, FVC, DLCO), HRCT findings, PASP post-CYC.	Overall mortality: 19.8% (17/86), PAH group: 50% mortality vs. 12% without PAH (P = 0.001), ILD did not affect mortality (19% with ILD vs. 21% without; P = 0.99)	PAH was the strongest predictor of mortality, independent of ILD. CYC stabilized ILD (TLC/DLCO unchanged) but did not prevent PAH progression (PASP increased; P = 0.015). DLCO was significantly lower in PAH patients (P = 0.0015) and correlated with mortality.	PAH (HR = 4.09, P = 0.007), Older age at diagnosis (HR = 1.057, P = 0.02), Low DLCO (P = 0.007), FVC:DLCO ratio ≥2 associated with PAH (P < 0.0001) and mortality (P = 0.03)	PAH progression despite CYC therapy, no CYC benefit on PASP reduction, Non-significant factors: ILD extent, cancer, cardiac or renal involvement
Bauer et al., 2013 [35]	To determine the influence of ILD on morbidity and mortality in SSc patients compared to those without ILD.	Cohort study; N = 64 incident SSc patients. 89% female; median age: 49.1 years.	Treatments included immunosuppressants, supplemental oxygen, and vasoactive agents (not specified). No patients underwent lung transplantation.	Mortality, hospital admissions, and organ involvement (ILD, PAH, CKD, CHF).	Median survival: 22.9 years, Kaplan-Meier curves and Cox regression used to identify risk factors, ILD, PAH, and CKD significantly associated with reduced survival (P < 0.05)	ILD, PAH, and CKD are each linked to poor outcomes. ILD was present in 30% of cases and contributed to one death. PAH was the leading cause of death (7/17 deaths).	ILD, PAH, and CKD were all associated with increased mortality risk (P < 0.05). Older age was also a poor prognostic factor. No significant effect from CHF or smoking history	PAH contributed to 7 deaths; ILD to 1 death, CHF (42%), and smoking had no significant mortality impact
Jang et al., 2023 [36]	To identify risk factors for mortality and compare clinical characteristics in patients with SSc-ILD.	Retrospective cohort study; N = 106 SSc-ILD patients. 60 with limited disease, 46 with extensive disease. Mean age: 51.9 years; 86.8% female. Median follow-up: 92.8 months.	Steroids (76.4%), azathioprine (23.6%), cyclophosphamide (7.5%), mycophenolate mofetil (7.5%), methotrexate (6.6%), Antifibrotic: D-penicillamine (36.8%)	Mortality, disease progression (FVC decline >10% or FVC 5–9% + DLCO decline >15%), pulmonary hypertension (RVSP >40 mmHg), GAP score.	Overall mortality: 19.8% (21/106), Extensive group: 32.6% mortality vs. 10.0% in limited group (P = 0.011), PH more frequent in extensive group: 43.5% vs. 16.7% (P = 0.009)	The extensive disease group had higher ESR (P = 0.003), PH prevalence, and mortality. FVC decline occurred in both groups: 15%–20% (1st year), 8%–10% (2nd year).	Older age (OR = 1.063, P = 0.009), Lower FVC (OR = 0.958, P = 0.004), Extensive disease (OR = 7.802, P = 0.001)	Causes of death: pneumonia (n = 6), malignancy (n = 7), ILD acute exacerbation (n = 2), PH, and extensive disease associated with higher mortality
Knarborg et al., 2022 [37]	To investigate the incidence and prevalence of SSc and its association with ILD.	Retrospective cohort study based on a national registry. Female predominance: 75.5%. Mean age at SSc diagnosis: 56.7 years (non-ILD) vs. 57.7 years (SSc-ILD).	Patients were managed through centralized care at specialized centers with multidisciplinary follow-up. ILD screening included HRCT and annual pulmonary function tests.	Primary: SSc and SSc-ILD incidence and prevalence. Secondary: Demographic and regional predictors of SSc-ILD.	Incidence stable: 2.9/100,000 annually, Prevalence increased: 14.1–16.5/100,000 (2000–2008) to 17.9–19.2/100,000 (2009–2016), SSc-ILD prevalence: 14.7% (lower than prior estimates).	Stable SSc incidence but rising prevalence suggests improved diagnostics and survival. SSc-ILD prevalence was lower than previously reported (18.8–52.3%). Higher male representation in the SSc-ILD group (30.9% vs. 23.4%, P = 0.008).	Male sex (HR = 1.75, P = 0.01) , Age 41–50 (HR = 1.81, P = 0.02), North Denmark residency (HR = 1.95, P = 0.02), Non-significant: marital status, education level	No treatment complications reported; no clinical data on ILD severity or SSc subtype distribution.

Prevalence of PH in SSc-ILD: The prevalence of PH among patients with SSc-ILD is significant and has been consistently documented across multiple studies. In a prospective cohort study by Young et al. (2019), PH was confirmed in 31.2% of SSc-ILD patients via right heart catheterization, highlighting a substantial burden within this population [17]. Similarly, Moinzadeh et al. (2024), analyzing a large cohort from the German Network for Systemic Sclerosis (DNSS), reported a 6.1% prevalence of combined ILD and PH, with this group demonstrating the worst five-year survival at 79.1% [23]. The study of Amikishiyev et al. (2025) found that SSc patients with ILD and PH had a much higher death rate, where ILD was shown to be a factor on its own (odds ratio (OR) = 5.7, p = 0.008) [25]. At the same time, Fairley et al. (2023) in the Australian Scleroderma Cohort Study found that 7% of people developed both PAH and ILD (PAH-ILD), and those individuals had the worst survival, with a hazard ratio (HR) of 5.68, being 5.68 times more likely to die than SSc-only patients (p < 0.001) [27]. Mathai and colleagues demonstrated in their 2009 paper that ILD-PH survival, measured over three years, was 39%, much less than the survival rate for isolated PAH. They suggest that PH occurs frequently in SSc-ILD, which is dangerous and leads to worse results because close monitoring and effective treatments are needed [29].

Survival rates of SSc-ILD without PH: It is always clear that patients with SSc-ILD not complicated by PH have a more hopeful outcome than patients with both ILD and PH. Moinzadeh et al. (2024) presented an extensive group of data, showing that survival in patients without PH and SSc-ILD lasted five years in 92.8% of them, while only 79.1% survived in those with SSc-ILD and PH [23]. Young et al. (2019) found that 97% of their SSc-ILD patients survived for three years, and this was primarily seen in those who did not have PH [17]. Noviani et al. (2019) further reinforced this, showing that SSc patients with isolated ILD had a five-year survival rate of 90% from the time of SSc diagnosis and 87.2% from ILD diagnosis, both markedly higher than the survival rates observed in those with concomitant PH. Their study stratified patients into isolated ILD, PAH, ILD-PH, and no/mild pulmonary involvement and found that isolated ILD conferred the second-best prognosis after the no/mild group [28].

Chan et al. (2025) also provided insight into mortality within the SSc-ILD population. In their retrospective multicenter study, the overall mortality rate for the ILD subgroup was 29.7%, compared to 18.8% in the non-ILD SSc group. Although this figure reflects long-term mortality and includes all causes, it further illustrates that even within the ILD cohort, outcomes are considerably worse only when PH develops [26]. Additional support comes from Fairley et al. (2023), who reported that patients with limited ILD only did not experience a statistically significant increase in mortality compared to SSc-only patients (HR = 1.52, P = 0.074), again emphasizing that mild to moderate ILD without PH does not substantially worsen long-term survival [27]. SSc-ILD without PH is associated with relatively high survival rates, with three- to five-year survival typically exceeding 87%-93% and substantially better outcomes compared to SSc patients with concomitant PH.

Survival rates of SSc-ILD without PH: Survival in SSc-ILD with PH is markedly worse than in patients without PH, as demonstrated across multiple studies. In a pivotal prospective cohort by Young et al. (2019), SSc-ILD patients with PH had a three-year survival rate of 91% after PH diagnosis, lower than the 97% survival in the total SSc-ILD group. Significantly, the presence of PH was associated with older age at ILD onset, lower diffusing capacity of the lung for carbon monoxide (DLCO), and increased need for supplemental oxygen, all indicative of more severe disease and higher mortality risk [17]. The Moinzadeh et al. 2024 study, encompassing over 3,200 patients from the DNSS, found that SSc-ILD patients with PH (ILD-PH subgroup) had the worst five-year survival rate at 79.1%. This was significantly lower than ILD patients without PH (92.8%) and those without pulmonary involvement (96.4%). Multivariable analysis confirmed ILD-PH as the subgroup with the highest mortality risk (HR = 5.3, p < 0.001) [23].

In the Fairley et al. 2023 study from the Australian Scleroderma Cohort, the combined PAH-ILD group had the poorest survival among all systemic sclerosis phenotypes, with an HR of 5.68 (P < 0.001) for mortality compared to the SSc-only group. Mortality in this group reached 50.5%, significantly higher than in patients with ILD alone or PAH alone [27]. Mathai et al. (2009) found that patients with ILD and PH had a poor long-term outcome: survival fell from 82% after one year to 46% after two years and to 39% after three years. Compared to those with PAH alone (survival of 64% at three years after treatment), this combination proved worse for patients [29]. Additionally, Noviani et al. (2019) found that just four years after their diagnosis, patients with both SSc and PH-ILD had a 77% survival rate, which was lower than other groups, very much like the ILD-PH group. Among all patients, those with ILD-PH had the highest chance of death (HR = 3.77, p < 0.001) [28].

Chauvelot and his team reported that the survival rates in the PH-ILD subgroup of SSc patients at one, two, and three years were 81.8%, 57.6%, and 41.5%, respectively, which were not as good as those seen in the isolated PAH subjects. There is an independent tie between ILD and lower survival, with immunosuppressive treatment being effective but only moderately so (HR = 2.11, p = 0.01), and immunosuppressive therapy improved the results, but not very significantly [31]. SSC-ILD, when present with PH, is known for a reduced life expectancy, as only half or fewer of patients often survive after three years, and survival rises above 80% in very few individuals at five years. Such data demonstrate that recognizing this genetic trait early and giving aggressive treatment and close supervision to patients with it are very important.

Prognostic factors: In SSc-ILD, especially those with PH, studies have found several reliable signs of a poor prognosis. Everyone agrees that PH causes the biggest and most dangerous prognostic issue. According to Moinzadeh et al. (2024) and Fairley et al. (2023), patients with SSc-ILD and PH had a much higher risk of death, with HRs of 5.3 and 5.68 [23, 27]. Older age at diagnosis has been consistently associated with worse survival, as shown in studies like Mathai et al. (2009) and Launay et al. (2013) [21, 29]. Similarly, male sex has been repeatedly identified as a negative prognostic indicator in cohorts such as those by Kolstad et al. (2018) and Moinzadeh et al. (2024) [20, 23].

Functional lung impairment, notably reduced DLCO and forced vital capacity (FVC), is a powerful predictor of mortality. Guler et al. (2018) and Trad et al. (2006) highlighted that a lower DLCO and an elevated FVC/DLCO ratio (≥2) are associated with PH development and death [30]. The extent of ILD also plays a role; Jang et al. (2023) and Knarborg et al. (2022) found that patients with extensive ILD (defined by FVC <70% or >20% HRCT involvement) had significantly higher mortality (OR = 7.8) [36, 37]. Hemodynamic markers such as elevated pulmonary vascular resistance (PVR) and low cardiac output were also predictive of poor outcomes, with Mathai et al. (2009) and Amikishiyev et al. (2025) confirming these as independent risk factors [25, 29].

Cardiac involvement, including right ventricular dysfunction and pericardial effusion, was associated with worse outcomes in Guillén-Del-Castillo et al. (2022) [24]. Treatment-related variables also influence prognosis: Amikishiyev et al. (2025) found that dual or triple PH-targeted therapy significantly improved survival compared to monotherapy, while Chauvelot et al. (2020) reported reduced mortality with immunosuppressive therapy in PH-ILD patients [25, 31]. Conversely, delayed initiation of treatment after pulmonary function decline was associated with irreversible damage and higher mortality, as per Hoffmann-Vold et al. (2020) [18]. Inflammatory biomarkers, including elevated ESR and CRP, were linked to disease progression and death in studies by Chan et al. (2025) and Hoffmann-Vold et al. (2021) [18, 26]. Greater chances of a good outcome were linked to ANA-centromere positivity in Fairley et al. 2023, while anti-Scl-70 tended to correlate with more serious illness [27]. In general, the results show that prognosis in SSc-ILD is influenced by multiple factors such as PH status, lung function, blood pressure measurements, demographics, and treatment effectiveness.

Mortality causes: Several studies have shown that the leading reasons for death in SSc-ILD with and without PH are problems involving the lungs, most often related to PH, while infections and involvement of other organs come after. In patients affected by SSc-ILD, deaths from PH are mostly caused by problems with the respiratory and cardiopulmonary systems. They reported that among deaths in the ILD-PH group, respiratory failure led to almost three-quarters, while right heart failure was the main cause of death in nearly two-thirds of the isolated PAH group [29]. Le Pavec et al. (2011) found that respiratory failure (48%) and right-sided heart failure (30%) were the most frequent causes of death in PH-ILD patients, with worsening oxygenation and renal impairment serving as predictors [33]. Amikishiyev et al. (2025) also found that cardiopulmonary failure (26%) and infections (26.1%)-including COVID-19-were the most common causes of death, followed by malignancy (13%) [25]. Notably, patients with ILD and PH who died had significantly worse pulmonary function (lower FVC), higher estimated pulmonary artery systolic pressure (ePASP), and lower cardiac output.

In broader SSc cohorts, Fairley et al. (2023) observed that patients with PAH-ILD had the highest mortality rate (50.5%), with infections identified as a major contributor [27]. Chan et al. (2025) also noted that in the ILD subgroup, pneumonia was the leading cause of death, accounting for 63.6% of deaths, and that mortality was higher in those with progressive ILD and PH [26]. Noviani et al. (2019) confirmed that among SSc patients with ILD-PH, PAH and ILD together accounted for 34% of deaths, again underscoring pulmonary complications as the leading threat [28]. In contrast, SSc-ILD patients without PH tend to have lower mortality rates and a broader distribution of death causes, including malignancy, infections, and non-pulmonary organ complications. However, these are much less prevalent compared to the dominant respiratory causes in the PH subgroup. The primary causes of mortality in SSc-ILD with PH are respiratory failure and right heart failure, driven by the dual burden of fibrotic lung disease and pulmonary vascular involvement. Infections and malignancies are also essential contributors, particularly in immunosuppressed patients. Early identification of PH and proactive management of lung and cardiac complications are thus essential to improving survival.

Discussion

The presence of PH in patients with SSc-ILD is consistently associated with significantly worse survival outcomes, as demonstrated across multiple studies. Bauer et al. (2013) highlight that SSc-ILD patients without PH tend to have favorable prognoses, reporting three-year survival rates as high as 97% and five-year survival rates ranging from 87% to 93% [35]. In stark contrast, SSc-ILD patients with PH experience markedly reduced survival, with Mathai et al. (2009) reporting a three-year survival rate as low as 39% and Moinzadeh et al. (2024) noting a five-year survival rate of only 79% for this group. This substantial survival disparity underscores the prognostic impact of PH in SSc-ILD populations [23,29].

Further supporting these findings, Fairley et al. (2023) quantified the increased mortality risk associated with PH and ILD overlap by reporting an HR of 5.68 for patients with both conditions compared to those with SSc alone [27]. Yatsyshyn et al. (2024) corroborated the detrimental effect of PH, observing a three-year survival rate of just 56% in a broader SSc-PH cohort that included both ILD and non-ILD cases. Although the inclusion of non-ILD patients limits direct comparison, the findings still reflect the overall negative impact of PH on survival in systemic sclerosis [38].

Lee and Bull (2019) provide additional context by demonstrating that cardiopulmonary complications, specifically ILD and PH, have emerged as the leading cause of death in Ssc in the modern treatment era [39]. This trend reinforces the conclusions of more recent studies, such as Le Pavec et al. (2011), which identified the PH-ILD combination as the most lethal SSc phenotype. Despite variations in cohort design, timeframes, and outcome measures across studies, the collective evidence consistently highlights PH as a critical factor driving mortality in SSc-ILD, emphasizing the need for vigilant screening and aggressive management in this high-risk subgroup [33].

The reviewed literature identifies various prognostic factors that significantly influence survival in patients with SSc-ILD. Smith, Marjenberg, and Volkmann (2025) indicated that the presence of PH consistently emerges as the most potent predictor of poor outcomes, with multiple studies reporting hazard ratios exceeding five for mortality in patients with combined PH and ILD compared to those without PH [40]. Cacciapaglia et al. (2023) to PH, several other adverse prognostic indicators have been reported, including older age at diagnosis, male sex, extensive ILD on imaging, reduced DLCO, and hemodynamic markers such as elevated PVR and low cardiac output [32]. Chauvelot et al. (2020) indicated that cardiac involvement, particularly right ventricular dysfunction, further compounds the risk and is associated with significantly worse outcomes [31].

Noviani et al. (2019) demonstrated that a DLCO of less than 55% predicted significantly poorer survival in patients with limited systemic sclerosis and ILD, reinforcing similar observations made by Guler et al. (2018), who also highlighted the prognostic utility of DLCO in SSc-ILD [28, 30]. Pasarikovski et al. (2016) added another dimension by showing that the rate of ILD progression itself is a strong predictor of mortality, a finding that aligns with Moinzadeh et al.'s 2024 observation of high mortality in patients with progressive ILD and concurrent PH [23, 41]. Hemodynamic parameters such as PVR and cardiac output, emphasized in Mathai et al. (2009), have also been validated in other connective tissue disease-associated PAH cohorts, including the study by Yuan et al. (2022), which found these measures to be universally predictive of outcomes across various SSc-related pulmonary complications [29, 42].

The causes of mortality in patients with SSc-ILD differ notably depending on the presence of PH. In SSc-ILD patients with PH, respiratory failure and right heart failure dominate as leading causes of death, accounting for up to 75% of mortality in some cohorts. Infections, particularly pneumonia and COVID-19, also contribute significantly, especially among those receiving immunosuppressive therapy. In contrast, SSc-ILD patients without PH exhibit a broader and less severe spectrum of mortality causes, including malignancies and non-pulmonary organ complications, reflecting a more systemic but less acutely life-threatening disease course.

Studies on SSc-ILD with PH reveal a unique pathophysiology where fibrosis leads to ventilatory impairment and PH induces circulatory failure, leading to worse survival compared to isolated PAH or ILD alone. Modern SSc mortality is increasingly driven by combined cardiac and pulmonary involvement. Treatment studies show that PH-targeted therapies and immunosuppressants offer modest survival benefits in ILD-PH, but these improvements are often limited when initiated late. Early immunosuppression slows ILD progression, suggesting that early, combined therapy may be more effective in preventing irreversible damage. The HR for mortality in ILD-PH patients is 5.68, highlighting the amplified risk in this phenotype.

Clinical Implications

The evidence underscores the critical need for early identification and aggressive management of pulmonary hypertension in patients with SSc-ILD. The high mortality associated with the ILD-PH, combining ventilatory and circulatory failure, warrants a shift from reactive to proactive care models. Regular screening for PH in SSc-ILD patients using echocardiography and right heart catheterization should be standard, particularly in those with worsening functional capacity or oxygenation. Moreover, a combination of immunosuppressive therapy and PH-targeted treatment may offer the most benefit, especially when initiated early in the disease course. Tailored treatment strategies based on phenotypic differentiation (e.g., ILD-dominant vs. PH-dominant) are essential to improve outcomes and reduce mortality.

Limitations and Future Directions

Despite valuable insights, several limitations remain across the reviewed studies. Many analyses are retrospective, cohort sizes are often small, and the heterogeneity in patient populations (e.g., degree of fibrosis, PH severity, treatment regimens) complicates cross-study comparisons. There is also a lack of randomized controlled trials (RCTs) specifically addressing the efficacy of combination therapy in ILD-PH. Future research should focus on prospective, multicenter RCTs to evaluate early intervention strategies and the optimal timing and combination of therapies. Biomarker discovery for early PH detection and personalized treatment algorithms are promising areas for future investigation. Furthermore, longitudinal studies are needed to better define the natural history of SSc-ILD with and without PH, particularly in the era of evolving therapeutics such as antifibrotic agents and novel vasodilators.

## Conclusions

SSc-ILD patients with coexisting PH face significantly higher mortality rates than those without PH, primarily due to compounded respiratory and cardiac failure. This dual burden of fibrosis and vascular disease demands early recognition and phenotype-specific interventions. Although current therapies provide only modest survival benefits, the potential for improved outcomes through early combination treatment is evident.
